# Enhancing YOLOv8s with MHSA and GA for robust caries detection on periapical radiographs

**DOI:** 10.1186/s12903-026-08806-5

**Published:** 2026-06-11

**Authors:** D Meghana, A Manimaran

**Affiliations:** Department of Mathematics, School of Advanced Sciences, VIT-AP University, Beside AP Secretariat, Amaravati, 522241 Andhra Pradesh India

**Keywords:** Dental caries detection, Intraoral periapical radiographs, YOLOv8, Multi-head self-attention, Genetic algorithm

## Abstract

**Background:**

Early identification of dental caries on Intraoral Periapical Radiographs is clinically important, yet automated detectors often show unstable performance across datasets due to subtle lesion appearance and limited generalization.

**Methods:**

We propose an attention-augmented YOLOv8s detector that inserts two Multi-Head Self-Attention (MHSA) blocks in the deeper backbone and couples this architecture with a compact Genetic Algorithm (GA)–based schedule search to stabilize optimization. Experiments were conducted on a curated dataset collected at Sibar Institute of Dental Sciences, Guntur (1,887 radiographs), standardized and quality screened, and split into 80% for training, 10% for validation, and 10% for testing.

**Results:**

Using identical augmentations and a fixed inference operating point, the proposed YOLOv8s+MHSA+GA achieves Precision 0.98, Recall 0.94, F1 Score 0.96, mAP@0.5 0.97, and mAP@0.5:0.95 0.94. Relative to the next-best baseline (per metric), this corresponds to +0.21 mAP@0.5 (0.97 vs 0.76), +0.61 mAP@0.5:0.95 (0.94 vs 0.33), and +0.26 Recall (0.94 vs 0.68). We further report Wilson 95% confidence intervals for precision and recall to quantify statistical uncertainty.

**Conclusions:**

These results indicate that deeper-stage self-attention, paired with principled schedule selection, delivers clinically meaningful gains in sensitivity and localization tightness while preserving the efficiency advantages of a one-stage detector, supporting routine AI-assisted diagnosis.

## Introduction

Dental caries of permanent teeth is one of the most prevalent chronic diseases worldwide and a major contributor to the global oral health burden. Beyond localized dental destruction, accumulating evidence links untreated caries with systemic conditions including cardiovascular disease, diabetes, and respiratory illnesses, highlighting its broader health significance. The burden is disproportionately higher in low- and middle-income countries where limited access to oral healthcare, inadequate hygiene practices, and dietary factors exacerbate disease progression. These disparities underscore the urgent need for effective, accessible diagnostic tools that enable early detection and timely intervention, ultimately improving population health equity [1].

Artificial Intelligence (AI) enables machines to perform tasks traditionally requiring human expertise, with Machine Learning (ML) allowing systems to learn from data rather than explicit programming. Deep Learning (DL), particularly through Convolutional Neural Networks (CNNs), has demonstrated exceptional performance in medical image analysis by learning hierarchical feature representations directly from raw data. CNN-based approaches have shown remarkable success across medical diagnostics, treatment planning, and disease monitoring, often achieving expert-level performance in classification, detection, and segmentation tasks [2].

In dentistry, DL has been increasingly applied to orthodontic landmark detection, endodontic lesion identification, periodontal assessment, and caries analysis [3]. Dental caries is traditionally diagnosed through visual-tactile examination supported by radiographs; however, early lesions are frequently missed, and inter-examiner variability remains high. Missed enamel lesions can progress silently to dentin and pulpal involvement, shifting care from preventive to invasive procedures [4]. Systematic reviews report that DL systems trained on intraoral radiographs achieve clinically acceptable accuracy with strong specificity for approximal caries and can function as reliable assistive tools under expert supervision [5]. These systems can highlight suspicious regions, reduce missed early lesions, and improve diagnostic consistency, particularly in resource-limited settings.

This study focuses on Intraoral Periapical Radiographs (IOPA) rather than bitewings. IOPA are widely used in routine practice because they visualize the crown, root, and periapical structures at low cost and radiation dose. However, they present greater technical challenges for automated detection: lesions are often subtle, low in contrast, and confounded by restorations, projection variability, and overlapping anatomy. In contrast, bitewings are optimized for proximal caries with standardized geometry. Despite their routine use, IOPA remain comparatively underexplored in DL literature, motivating the need for robust detection methods tailored to this more challenging modality.

Among CNN detectors, the YOLO family has shown strong suitability for dental radiographs due to its ability to localize and classify lesions in a single step with real-time efficiency. Recent studies using advanced YOLO variants on bitewing radiographs report diagnostic performance approaching or exceeding that of dentists [6]. Comparative analyses confirm the robustness of YOLO architectures for radiographic caries detection [7]. These findings establish YOLO as a reliable backbone for automated dental image analysis.

However, most prior work relies on convolution-only architectures and predominantly evaluates bitewing images. CNNs inherently possess limited receptive fields and may fail to capture long-range contextual relationships, which are crucial in radiographs where lesions occur within complex anatomical surroundings. Transformers, particularly Swin Transformer–based designs, address this limitation using Multi-Head Self-Attention (MHSA) to model global context and multi-scale relationships. Hybrid CNN-Transformer designs have shown improved performance for dental imaging tasks by combining boundary detection with global relational understanding [8].

Recent work further demonstrates that embedding MHSA within convolutional detectors significantly improves dental radiograph analysis. Incorporating MHSA into YOLOv4 enhanced its ability to emphasize diagnostically relevant regions, improving Precision, Recall, and F1 Scores [9]. These results confirm that attention mechanisms can effectively complement convolutional feature extraction. Building on this evidence, the present study integrates deep-stage MHSA into YOLOv8s, a more advanced and efficient detector than YOLOv4, featuring improved feature fusion and decoupled heads. This hybrid design aims to retain the efficiency of one-stage detection while enhancing contextual feature modeling critical for subtle caries in IOPA images.

Decision-support systems are particularly valuable when they reduce missed early lesions and inter-reader variability. Prior studies show AI assistance improves sensitivity while maintaining acceptable specificity, functioning as a reliable second reader. Motivated by this, we couple MHSA insertion with a Genetic Algorithm (GA)–based schedule search to stabilize training after attention integration and to improve recall over a strong YOLOv8s baseline. We report Precision, Recall, F1 Score, mAP@0.5, and mAP@0.5:0.95 to evaluate both detection performance and localization tightness.

Prior studies report promising results for object detection frameworks on bitewings. Kunt et al. demonstrated strong ensemble performance across detectors [10]. Tichy et al. showed AI matching expert dentists and outperforming novices [11]. Other large-scale studies with YOLO variants report competitive precision but note sensitivity to artefacts, class imbalance, and label noise [5, 12]. Two-stage and segmentation frameworks such as Faster R-CNN and U-Net have also improved sensitivity [13, 14], while transformer-assisted Mask R-CNN approaches show promise for secondary caries and lesion staging [15–17]. Nevertheless, most studies rely on bitewing images, single-center datasets, and heterogeneous protocols, limiting comparability and leaving IOPA underexplored [7, 18].

Despite promising progress, current state-of-the-art methods for automated caries detection still have several important limitations. First, many studies are based primarily on bitewing radiographs, whereas IOPA remain comparatively underexplored despite their routine clinical use and greater anatomical complexity. Second, several existing models show reduced sensitivity for small, subtle, or early lesions, which are often the most clinically important to detect. Third, many studies, including the present work, rely on single-center datasets, highlighting the need for future validation across multiple institutions, imaging devices, and acquisition protocols to further establish generalizability. Finally, while transformer-based or hybrid architectures can improve contextual modelling, they may introduce additional computational cost, emphasizing the importance of designing methods that enhance detection performance while maintaining practical efficiency for real-time clinical use.

The main contributions of this work are as follows:

- Deep-stage MHSA for subtle periapical caries: We enhance YOLOv8s by inserting two MHSA blocks in the deepest backbone stages to better capture long-range context while keeping the standard YOLOv8s design otherwise unchanged.
- Compact GA schedule search for stable training: To stabilize optimization after attention insertion, we introduce a small-budget GA to search a compact set of schedule-related hyperparameters (e.g., learning rate, weight decay, optimizer, mosaic probability, input resolution, batch size) using a fixed validation protocol, and then lock the best configuration for the final long run.
- Systematic ablations under a fixed protocol: We report a controlled progression (YOLOv8s *→ * YOLOv8s+MHSA *→ * YOLOv8s+GA *→ * YOLOv8s+MHSA+GA) using the same split and operating point, and evaluate with Precision, Recall, F1 Score, mAP@0.5 and mAP@0.5:0.95 to isolate the contributions of MHSA and GA and demonstrate their complementary effect on sensitivity and localization.

Early caries on IOPA is often subtle and low contrast, and is confounded by proximal contacts, restorations, and overlapping anatomy. We therefore place MHSA only in the deep backbone so early edge/texture extraction remains intact while higher-level features incorporate broader structural context. Because attention insertion can change feature statistics and gradient scales, we pair it with a compact GA schedule search to select a stable training regime under a fixed validation protocol, reducing reliance on manual tuning while preserving the efficiency advantages of a one-stage detector.

## Materials and methods

The dataset was prepared through a structured pipeline that began with preprocessing to support subsequent manual annotation and model training. After expert annotation, the finalized set was used to train a YOLO-based detector. Model performance was then quantified using established metrics. Detailed descriptions of each stage are provided below.

### Data collection

Data were gathered in collaboration with Sibar Institute of Dental Sciences, Thakkellapadu, Guntur, Andhra Pradesh, India an academic and clinical center that maintains a broad repository of diagnostic radiographs. Between November 2024 and February 2025, we assembled 2,452 intraoral periapical (IOPA) radiographs documenting confirmed dental caries. IOPA images are routinely used to detect, grade, and plan treatment for dental cavities, and thus provide an appropriate basis for this study.

All acquisitions were conducted under the guidance of dental specialists at the institute. These clinicians oversaw case identification, verification, and clinical categorization following standard diagnostic protocols. Images were transferred to study laptops via the hospital’s secure Wi-Fi with authorized access; the attending faculty supervised selections and ensured the correct clinical tags were applied.

Institutional Ethics Committee approval was obtained at Sibar Institute of Dental Sciences (SIDS) on 13 December 2024 prior to data collection, and informed consent was obtained where applicable. All radiographs were de-identified before analysis to ensure patient confidentiality. The intraoral periapical radiographs were acquired using an X-Mind DC dental X-ray unit (Acteon, France) in conjunction with a Dürr Dental VistaScan Mini Easy PSP scanner. Image acquisition was performed by trained dental professionals using the paralleling technique, which is the standard method for intraoral periapical imaging. Photostimulable phosphor (PSP) plates (Size 2) were used for image capture, with exposure times ranging from 0.12 to 0.32 seconds. The plates were scanned at a resolution of 20 line pairs per millimetre (lp/mm), and the resulting images were processed using VistaScan software and saved in PNG format for subsequent analysis.

### Data preprocessing

Robust training in medical imaging depends on careful preparation, as diagnostic quality can vary. We therefore implemented a comprehensive preprocessing workflow to standardize appearance and improve fidelity across the dataset. To protect patient confidentiality, we manually cropped every image to remove any identifying overlays (patient name, ID) commonly present at image corners. Anonymized images were then organized into a dedicated directory, ensuring that no personally identifiable information remained prior to downstream processing or model training.

We first screened image quality using Blind/Referenceless Image Spatial Quality Evaluator (BRISQUE), implemented in PyTorch via the PIQ library. In this implementation, BRISQUE is reported on a centered scale that can take negative values; lower is better, and values are not directly comparable to the canonical 0–100 index. This no-reference metric is suitable when ground-truth pristine images are unavailable. Lower BRISQUE values were interpreted as better perceived quality. Images were retained if they fell within the central quality band, and extreme outliers were excluded using interquartile range (IQR) analysis to remove unusually degraded or anomalously scored images on the centered scale. After quality filtering, 2,418 radiographs remained.

**Fig. 1:**
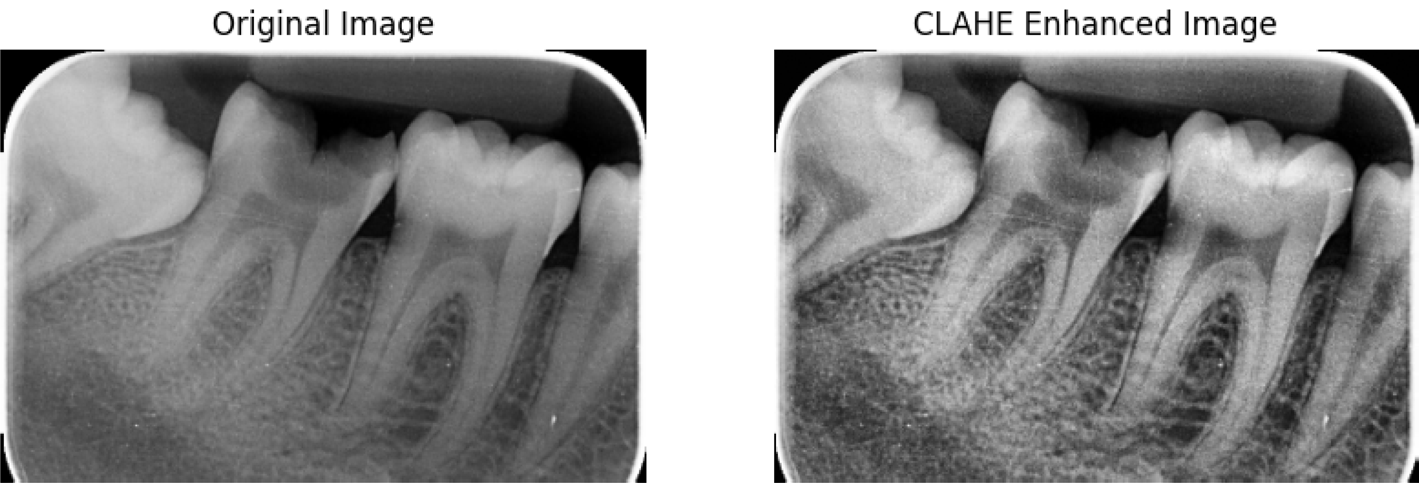
Original image and its enhancement

To improve local contrast without amplifying noise, we applied Contrast Limited Adaptive Histogram Equalization (CLAHE) with an 8*× *8 tile grid and a clip limit of 2.0. Visual comparisons Fig. 1 provide a clearer depiction of subtle details caries, such as early carious lesions and enamel boundaries after enhancement.

To increase variability and mitigate overfitting, we used the Albumentations library for data augmentation. All images were resized to 640*× *640 to match the YOLOv8s input size. We used horizontal and vertical flips (probability 0.5), rotations in multiples of 90°, small random rotations up to ±20°, and additional spatial transforms to reflect typical variations in radiographic positioning. These augmentations were designed to promote robustness across diverse clinical acquisition conditions.

**Fig. 2:**
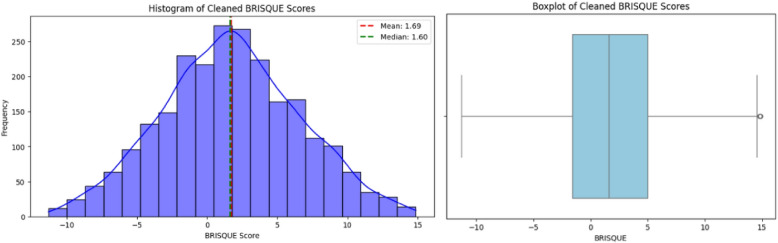
Distribution of BRISQUE scores

Finally, we summarized BRISQUE distributions on the centered scale after cleaning. The histogram shows a near-normal shape with a mean of 1.69 and median of 1.60, indicating consistency and low skew in the retained set. The accompanying boxplot Fig. 2 shows a narrow interquartile range with few outliers; most scores fall approximately between -12 and +15, supporting a high, uniform image quality after preprocessing.

### Data labelling and annotation

We conducted manual labelling in the Computer Vision Annotation Tool (CVAT), an open-source platform widely used for computer vision annotation. A dedicated task was created for dental caries detection, specifying the target class (Caries) and the bounding-box labelling model. The complete set of IOPA was uploaded to CVAT, and we drew rectangle bounding boxes around visually apparent carious lesions. Each image was examined carefully to position boxes precisely over the suspected regions, facilitating accurate lesion localization.

To ensure clinical fidelity, dental specialists reviewed all caries regions, as detailed below and bounding boxes were adjusted where necessary to reflect expert judgment. All annotations were initially performed by the first author using the Computer Vision Annotation Tool (CVAT) following a predefined protocol. The annotated images were subsequently reviewed and validated by Dr. S. Venkata Sai, a final-year postgraduate student in the Department of Conservative Dentistry and Endodontics, with six years of clinical experience and specialized training in caries diagnosis and radiographic interpretation. This expert verification ensured that the annotated regions were consistent with clinical findings observed in IOPA. Any discrepancies were resolved through discussion to maintain annotation consistency. Since the dataset was annotated by a single primary annotator with expert review rather than multiple independent annotators, formal inter-observer agreement statistics (e.g., kappa coefficient) were not assessed in the present study. Every annotated box was assigned the class index $\{0\}$ (caries). After labeling, the dataset was exported in YOLO detection format, which produces one text file per image containing the class index and normalized box coordinates in the order $[\texttt {class\_id}, x_{\text {center}}, y_{\mathrm{center}}, \mathrm{width}, \mathrm{height}]$. These label files are automatically paired with their corresponding images, enabling immediate integration into YOLO training pipelines.

The annotated intraoral periapical radiographs exhibit variability in anatomical coverage and lesion presentation. Depending on the presence of carious regions, images may contain either single or multiple bounding box annotations. Several radiographs include multiple lesions within the same tooth or across adjacent teeth, resulting in multiple annotated regions per image, while others contain only a single localized lesion. The bounding box sizes vary considerably, reflecting differences in lesion extent and stage of progression. Smaller bounding boxes typically correspond to early-stage proximal or enamel caries, whereas larger bounding boxes represent advanced dentinal involvement. This variation introduces natural scale diversity in the annotations. Due to the confined anatomical structure of teeth in periapical radiographs, bounding boxes are often closely spaced and occasionally in close proximity, creating a challenging multi-instance localization scenario for object detection models. This annotation distribution captures realistic clinical variability and provides a representative basis for evaluating multi-scale and multi-instance detection performance.

**Fig. 3:**
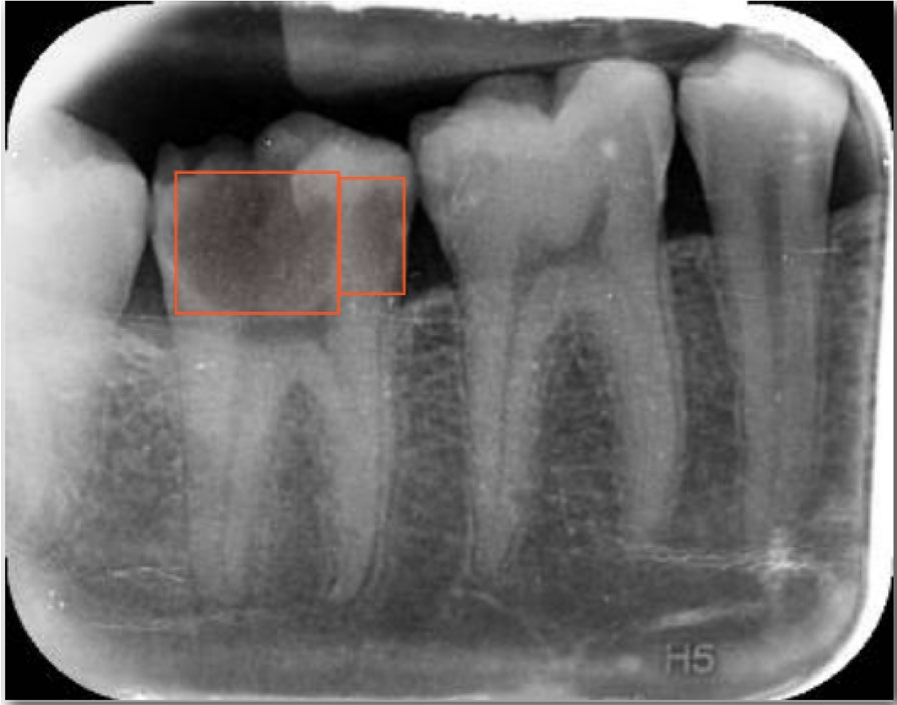
Annotated image

During quality control, repeated radiographs were removed to avoid redundancy and to prevent identical images from appearing across different data splits. The final curated dataset comprised 1887 images, partitioned into training (80%), validation (10%), testing (10%) splits. Patient-level identifiers were not consistently available; therefore, patient-wise splitting could not be performed. This is acknowledged as a limitation, as patient-level partitioning would further minimize the risk of potential data leakage and improve generalizability. Overall, CVAT provided a structured and efficient workflow that yielded high-quality, model-ready annotations for precise caries detection. Figure 3 shows a representative IOPA with annotated caries bounding boxes.

### YOLOv8s with MHSA-enhanced backbone

#### YOLOv8

YOLOv8 is an anchor-free, single-stage detector with a lightweight Cross-Stage Partial with Full Concatenation (C2f) convolutional backbone, a Path Aggregation Network/Feature Pyramid Network (PAN/FPN) neck for multi-scale fusion, and decoupled detection heads that predict classification logits and bounding boxes on three progressively coarser feature maps from fine to coarse resolution. The design emphasizes strong speed-accuracy trade-offs, efficient parameter usage, and stable training via modern choices (e.g., decoupled heads, better loss balancing), making it a practical baseline for clinical workflows where latency matters.

#### MHSA

MHSA computes content-adaptive global context by forming Queries (Q), Keys (K), and Values (V) from feature maps, applying scaled dot-product attention within each head, and aggregating results across heads. Unlike convolutions with fixed local receptive fields, MHSA allows long-range interactions between distant spatial locations, which is beneficial for subtle, low-contrast patterns common in radiographs. Lightweight instantiations using 1*× *1 projection, residual connections, and LayerNorm stabilize training while keeping compute overhead modest. A one-stage detector was preferred over two-stage architectures such as Mask R-CNN or Faster R-CNN because the intended application is clinically practical, near-real-time decision support, where low inference latency and efficient deployment are important. In our prior work [19], we evaluated a two-stage detector (Faster R-CNN) on a similar dataset and observed comparatively higher inference time and lower effectiveness in detecting subtle lesions. Based on these observations, YOLOv8s was selected due to its improved speed–accuracy trade-off and suitability for real-time clinical applications. Furthermore, the present work focuses on bounding-box based lesion detection. In simpler terms, the MHSA module enables the model to consider relationships between distant regions of the image, which is particularly useful in dental radiographs where caries may appear in complex anatomical contexts.

We build on the Ultralytics YOLOv8s detector and introduce a lightweight self-attention enhancement in the backbone to better capture long-range dependencies characteristic of subtle, low-contrast carious lesions. The base network is an anchor-free, single-stage design with a C2f convolutional stem, multi-scale fusion in the neck, and decoupled classification and regression heads, an attractive choice for clinical use because it offers a strong speed-accuracy trade-off and low latency on commodity GPUs, without changing the neck or heads. Our modification leaves the neck and heads untouched and concentrates extra representational capacity in the deeper backbone, where aggregating broader context benefits tooth morphology and lesion topology. In practical terms, this allows the model to better capture both local lesion details and broader contextual relationships within the radiograph.

### Integrated MHSA design, tensor compatibility, and PAN/FPN Neck with decoupled detection heads

We insert two MHSA blocks at the deepest backbone scale: MHSA-1 is placed after the final backbone C2f block, and MHSA-2 is placed after the SPPF module. This keeps the early, edge layers unchanged while enriching the higher-level semantic features that the neck layer fuses. We place MHSA only in the deeper backbone because early layers capture local edges, while deeper features benefit from long-range context across tooth morphology and proximal contacts, which is critical for subtle, low-contrast caries patterns in periapical radiographs. Each MHSA operates on an N*× *C*× *H*× *W tensor: queries, keys, and values are formed by 1*× *1 projection; multi-head scaled dot-product attention is computed; outputs are merged and projected back to C channels. Residual skips, a lightweight 1*× *1 Multi-Layer Perceptron (MLP) with Gaussian Error Linear Unit (GELU), and LayerNorm2d stabilize training. Implementation is done by wrapping (i) the final C2f block and (ii) the SPPF block as Sequential (block, TransformerBlock2d), so the Ultralytics graph and routing stay untouched.

**Fig. 4:**
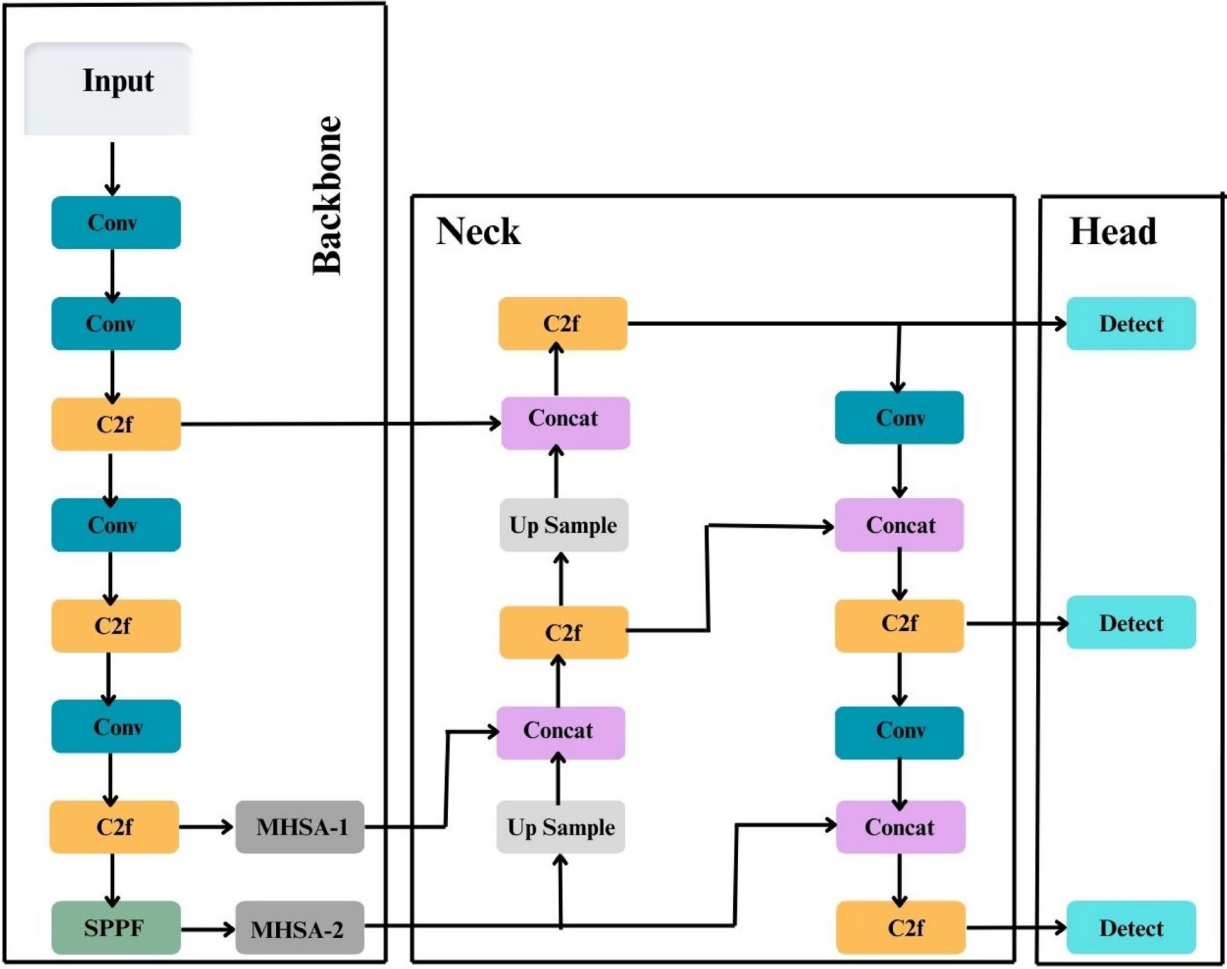
YOLOv8s with MHSA-enhanced backbone design

The neck remains a standard PAN/FPN. The coarsest feature coming out of SPPF is first refined by MHSA-2, then upsampled and concatenated with the medium-scale feature coming from the final C2f block (refined by MHSA-1). This fused map is passed through C2f, then upsampled again and concatenated with the finest backbone feature, followed by another C2f. A bottom-up refinement then downsamples the fine output, concatenates it with the medium output and applies C2f, then downsamples once more, concatenates with the coarse feature from the MHSA-2 branch, and applies C2f again. Three decoupled detection heads are finally attached to the fine, medium, and coarse outputs respectively. All losses, head definitions, and routing remain identical to YOLOv8s; observed gains therefore come from the added global-context modelling together with the GA-selected training schedule. Figure 4 illustrates the YOLOv8s backbone enhanced with the two MHSA blocks and the unchanged neck and decoupled heads.

**Fig. 5:**
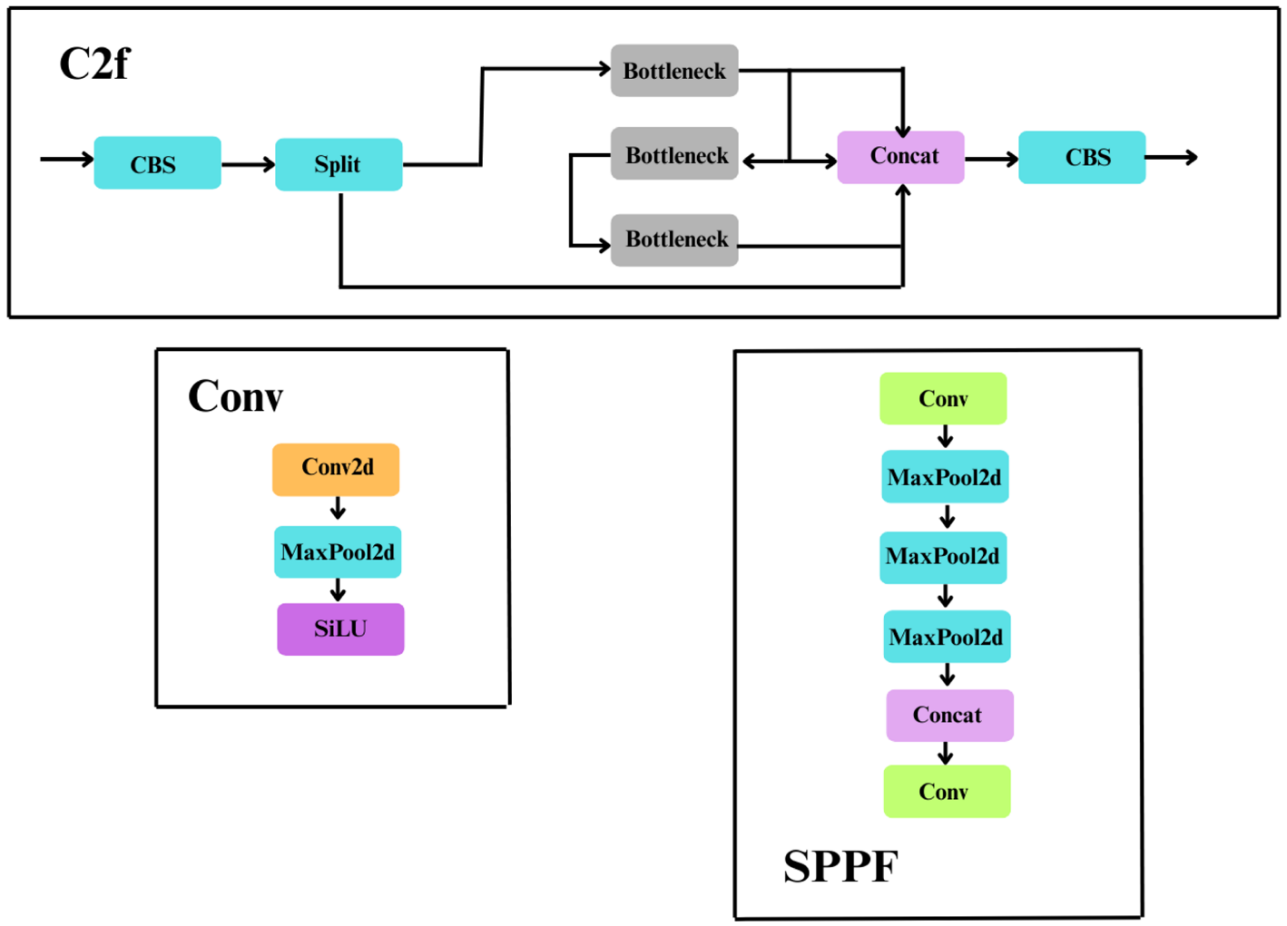
YOLOv8s core blocks

The Conv (Convolutional) block follows the standard YOLOv8s stem used throughout the backbone and neck: a 2-D convolution with the implementation’s default normalization and SiLU activation, chosen for its stable optimization and favorable latency. The C2f module realizes cross-stage partial aggregation: input features are split, processed through a small stack of bottlenecks, and concatenated back with the bypass branch, yielding efficient channel mixing with low parameter cost. The SPPF module approximates large-receptive-field pooling efficiently by stacking three max-pool operations at the same kernel size, concatenating the pooled maps with the skip, and finishing with a 1*× *1/3*× *3 convolution to mix channels. These schematics match the shapes/colors reused in Fig. 4 So that the architectural wiring and the building blocks can be read together. This module-level schematic is shown in Fig. 5.

**Fig. 6:**
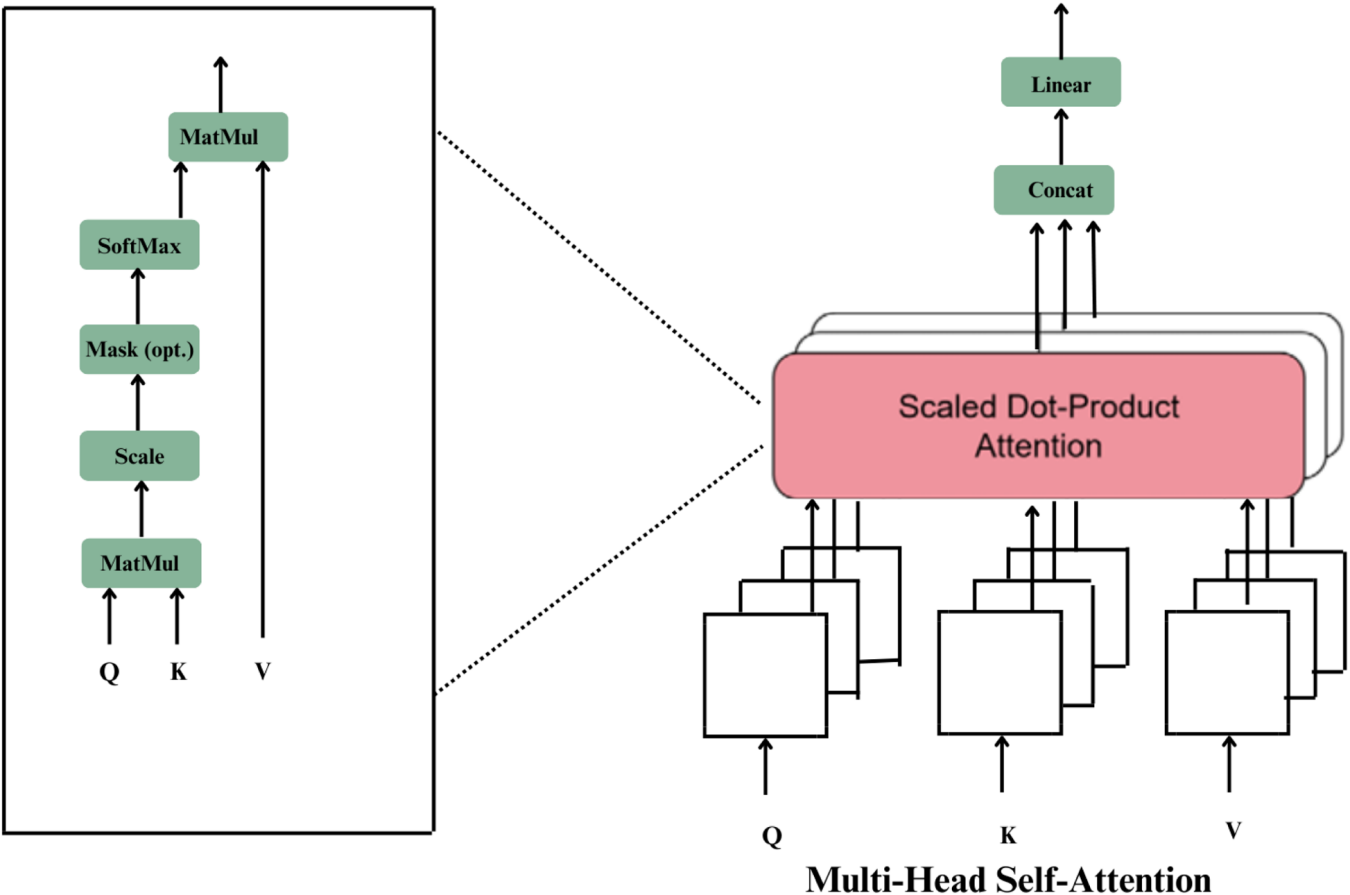
Multi-head self-attention

Each MHSA block operates directly on backbone feature maps $X \in \mathbb {R}^{N \times C \times H \times W}$. We flatten the spatial grid into *T = H · W* tokens and apply MHSA with *h* heads and per-head dimension *d = C/h*. Queries, keys, and values are produced by *1 × 1* projections (equivalently *1 × 1* convolutions) to form *Q*, *K*, and *V*, and attention weights are computed by scaled dot-product within each head and normalized by softmax; per-head responses are concatenated and projected back to *C* channels. Residual connections preserve gradient flow, a compact *1 × 1* MLP with GELU refines channel interactions, and LayerNorm2d (applied channel-wise in NCHW) stabilizes training. In our implementation, MHSA is attached by wrapping the target backbone blocks as Sequential (block, TransformerBlock2d), so tensor shapes and routing into the PAN/FPN neck remain unchanged and the decoupled detection heads are strictly identical to YOLOv8s. Therefore, inference-time overhead arises only from the added MHSA blocks (GA affects training only), while the overall detector structure and operating point remain unchanged. This MHSA cell is illustrated in Fig. 6.

### Training configuration

We initialized from yolov8s.pt and trained on the YOLO-formatted single-class caries dataset. To respect radiographic physics and reduce distribution shift, we apply deliberately gentle augmentations: small in-plane rotations (±1.5°), translations (2%), scaling (±10%), horizontal flip (20%), and light copy-paste (5%). We disable vertical flips, HSV color shifts, shear, perspective, and mixup; mosaic is used sparingly and automatically closed in the final epochs to stabilize convergence. A fixed seed ensures reproducibility of shuffling and initialization. We set warm-up to 3 epochs and retain Ultralytics default loss components and head formulations, while modestly relaxing the validation operating point (confidence = 0.20, Non-Maximum Suppression (NMS) IoU = 0.60) to favor small-lesion recall during model selection. Within this setup, we optimize a compact yet expressive set of hyperparameters and then lock them for a longer final run, aligning with best practice for fair model selection. Unless stated otherwise, the same augmentation and evaluation policy was used for all baseline models and ablations to ensure fair comparison.

### Genetic-algorithm (GA) hyperparameter optimization

The MHSA insertion subtly alters gradient scales and feature statistics. We tune the training regime via a small-budget GA that searches over the initial learning rate, weight decay, optimizer, mosaic probability, input resolution, and batch size. Each GA individual undergoes a short training of 18 epochs under the same augmentation family and warm-up, and we evaluate fitness using the Ultralytics validation routine with $\textrm{mAP}@0.5$ and $\textrm{mAP}@[0.5{:}0.95]$ at confidence *= 0.20* and NMS IoU *= 0.60*. Tournament selection, one-point crossover, and per-gene mutation balance exploration and exploitation across a population of eight for three generations, enough to find a good basin for this architecture while keeping compute modest. Because each fitness evaluation requires an end-to-end detector train/validate cycle, increasing population size or fitness epochs increases cost approximately linearly; we therefore chose a small population and short fitness horizon as a practical trade-off, and interpret GA here as compute-efficient exploration rather than a guarantee of global optimality. The best genome then defines the final configuration for a 400-epoch training with early stopping (patience *= 30*), after which we perform test-time evaluation using the same operating point to ensure comparability across baselines. In our GA, the maximized objective is

1 $$\begin{aligned} \underset{\theta \in \mathcal {H}}{\max } \textrm{mAP}_{0.5}(\theta ), \end{aligned}$$

where $\mathcal {H}$ is the discrete–continuous search space spanning $\{\texttt {lr0}, \texttt {wd}, \texttt {optimizer}, p_{\text {mosaic}}, \texttt {imgsz}, \texttt {batch}\}$; fitness equals the mean average precision at IoU *= 0.5* on the validation split computed over the single “caries” class.

### Final training and inference

Final training uses the GA-selected hyperparameters with the same gentle augmentations, three-epoch warm-up, and automatic mosaic closure to concentrate on real-image statistics in the tail of training. We retain Ultralytics decoupled heads and keep all detection head attachment points exactly as in the YOLOv8s baseline, leaving the inference graph unchanged except for the added attention in the backbone. For testing, we enable test-time augmentation in the validator to marginally stabilize predictions under small affine perturbations and evaluate at confidence = 0.20 and NMS IoU = 0.60, the same thresholds used during model selection, to avoid confounding due to shifting operating points. For reporting, we include Precision, Recall, F1 Score, and both mAP@0.5 and mAP@[0.5:0.95].

### Evaluation

**Fig. 7:**
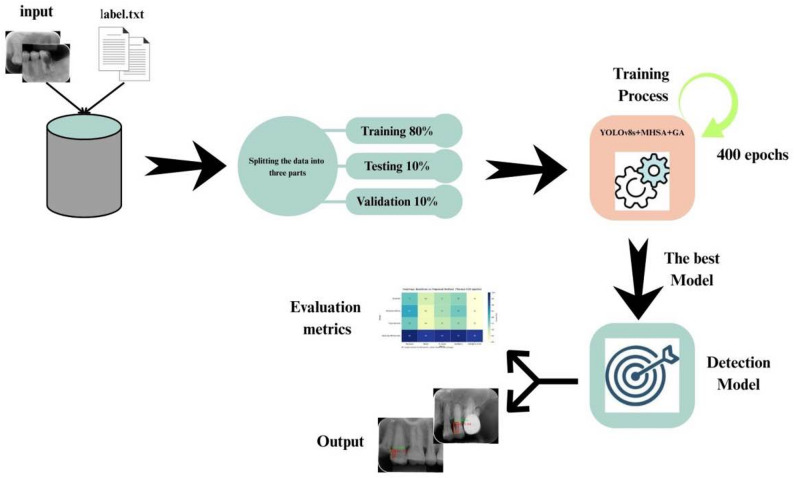
Flowchart of the proposed YOLOv8s+MHSA+GA-based dental caries detection pipeline

Performance is reported on the held-out test split using Precision, Recall, F1 Score, mAP@0.5 and mAP@[0.5:0.95]. Metrics were computed with the Ultralytics validation routine at a fixed operating point (confidence 0.20, IoU 0.60) across all variants: YOLOv8s, YOLOv8s+MHSA, YOLOv8s+GA, and YOLOv8s+MHSA+GA, to enable a fair comparison of operating thresholds; summary statistics account for the exact test set size evaluated for each model. Figure 7 Summarizes the end-to-end workflow, from data intake and split through training and model selection to quantitative evaluation outputs.

## Experimental results

### Selecting the baseline and final model

All detectors were trained on the same YOLO-formatted split, with *80%* for training, *10%* for validation, and *10%* for testing, using a fixed seed of 42. We applied identical radiograph-appropriate augmentations: small rotations of $\pm 1.5^{\circ }$, translations of *2%*, scaling of $\pm 10\%$, a horizontal flip of *20%*, and light copy-paste of *5%*, while vertical flips, HSV shifts, shear, perspective, and mixup were disabled. The Ultralytics pipeline was used across variants with warmup_epochs set to 3, close_mosaic set to 10, early-stopping patience set to 30, and evaluation at a fixed operating point with confidence *= 0.20* and NMS IoU *= 0.60*. Unless stated otherwise, long runs used 400 epochs, an image size within the range 512 to 768 (fixed at 640 for the non-GA baselines to ensure comparability), and the same gentle augmentations characteristic of IOPA. Evaluation on the held-out test split reports precision, recall, F1-score, and $\textrm{mAP}$ at IoU *= 0.5* using the Ultralytics validation routine under the same thresholds.

The baseline YOLOv8s was first trained without architectural modifications, establishing the convolution-only reference under our radiographic constraints. The resulting test metrics Precision 0.77, Recall 0.68, F1 Score 0.72, mAP@0.5 0.74, and mAP@0.5:0.95 0.33 showed a reasonable balance between localization accuracy and false-positive control at real-time speeds, but recall remained modest for a safety-critical screening context.

To test whether global context improves lesion sensitivity in low-contrast dental textures, we trained a backbone-augmented YOLOv8s+MHSA by inserting two lightweight MHSA blocks at the deepest backbone scale: MHSA-1 is placed after the final backbone C2f block and MHSA-2 is placed after the SPPF module, implemented by wrapping the final C2f and the SPPF modules as Sequential (block, TransformerBlock2d) so that tensor shapes and the neck and head routing remain unchanged. With the training schedule held fixed for 400 epochs and using identical augmentations and thresholds, Precision increased to 0.82 and mAP at IoU 0.5 rose to 0.76, while Recall remained at 0.64, F1 Score at 0.72 and mAP at IoU 0.5:0.95 was 0.32. This isolate-the-backbone comparison indicates that adding MHSA at the two deepest backbone stages strengthens class-conditional discrimination and improves ranking quality as reflected by mAP, but alone did not raise sensitivity on our dataset, consistent with the intuition that attention can focus features yet still requires tuned optimization to exploit the added capacity.

An optimization-only control, YOLOv8s+GA, was trained with the standard YOLOv8s backbone using hyperparameters selected by a compact GA that searched over learning rate, weight decay, optimizer choice, mosaic probability, input resolution, and batch size. The best configuration from the GA search was then fixed for a 400-epoch long run. This optimization, applied without MHSA, achieved Precision 0.76, Recall 0.64, F1 Score 0.70, mAP at IoU 0.5 of 0.73 and mAP at IoU 0.5:0.95 of 0.30. Gains over the baseline were negligible or slightly negative, suggesting that hyperparameter tuning alone does not resolve the dataset’s intrinsic difficulty when the feature extractor is strictly convolutional.

**Fig. 8:**
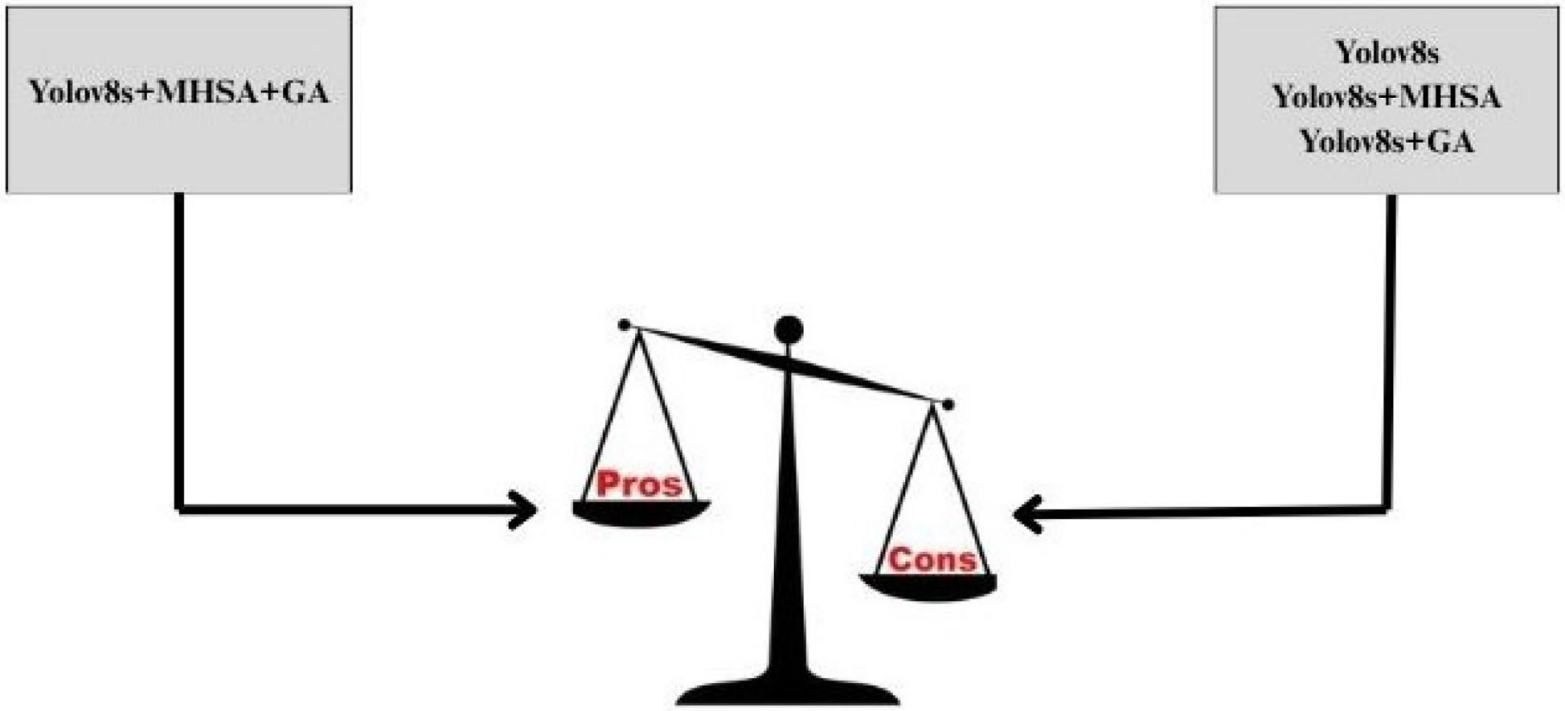
Schematic of the proposed model-comparison method

The proposed YOLOv8s+MHSA+GA combined a deeper-stage MHSA backbone with GA-selected training hyperparameters and was trained for a long time under the same early-stopping and augmentation regime. This model produced Precision 0.98, Recall 0.94, F1 Score 0.96, mAP at IoU 0.5 of 0.97, and mAP at IoU 0.5:0.95 of 0.94 on the test split, representing large improvements over all baselines. Relative to the convolution-only YOLOv8s, the combined system improved Precision by +0.21 or (+27.3%), Recall by +0.26 or (+38.2%), F1 Score by +0.24 or (+33.3%), mAP at IoU 0.5 by +0.23 or (+31.1%), and mAP at IoU 0.5:0.95 by +0.61or (+184.8%). The recall increase addresses the most critical clinical failure model of missed lesions without sacrificing precision, and the simultaneous rise in mAP@0.5 confirms better ranking/separation of positives across thresholds. Notably, mAP at IoU 0.5:0.95 rose from 0.33 with YOLOv8s to 0.94 with the proposed model, demonstrating accurate detections under stricter IoU thresholds and markedly tighter box localization. This improvement reflects more reliable ranking across operating points, supporting dependable thresholding and case triage in routine clinical use. As depicted in Fig. 8 the combined configuration aligns architectural capacity via deeper-stage MHSA with a compatible training regime using GA-selected hyperparameters, reducing missed lesions while maintaining very low false-positives rates and matching the quantitative gains. Clinically, this improvement is important because missed early lesions can progress to more severe conditions requiring invasive treatment. Higher recall therefore directly contributes to better preventive care and patient outcomes.

Taken together, these ablations support three conclusions. First, MHSA is beneficial but under-realized without matching optimization. On its own it raises precision and mAP yet does not lift recall on this dataset, indicating that deeper global context requires optimizer and regularizer settings that stabilize gradients and fully exploit the added capacity. Second, a genetic-algorithm search alone is insufficient, Tuning learning rate, weight decay, optimizer choice, mosaic probability, image size, and batch size yield only marginal gains when the backbones representational bias remains unchanged. Third, the combination is synergistic: MHSA provides long-range context aligned with radiographic textures, and the GA identifies a hyperparameter regime that enables those attention pathways to converge on a high-recall, high-precision optimum; the recall gain of 0.26 in absolute terms far exceeds what either ingredient contributed independently. These findings justify the central claim that a lightweight CNN detector, augmented with self-attention and paired with principled training-schedule optimization outperforms using only CNNs, only attention, or only optimization.

Qualitatively, predictions from the proposed model closely align with the annotations, with fewer partial boxes and fewer missed proximal lesions than the baselines, consistent with the higher recall and F1 Scores. Using the same operating point for evaluation, confidence threshold set to 0.20, and NMS IoU set to 0.60, the improved calibration of ranked outputs is reflected in the higher mAP at IoU 0.5, while the early-stopping patience and gentle augmentations preserved training stability across all runs. Because the neck and detection heads remained identical across variants, observed improvements can be attributed to two factors: first, backbone conditioning via MHSA; second, optimizer, regularization, and image-scale choices discovered by the GA, rather than to downstream architectural changes.

The staged evaluation YOLOv8s, YOLOv8s+MHSA, YOLOv8s+GA, YOLOv8s+MHSA+GA demonstrates that CNN + self-attention + optimization provides the strongest and most clinically relevant accuracy profile on IOPA, substantially reducing false negatives while maintaining very low false positives. This integrated design therefore offers a practical path toward real-time, trustworthy caries localization in routine workflows.

**Fig. 9:**
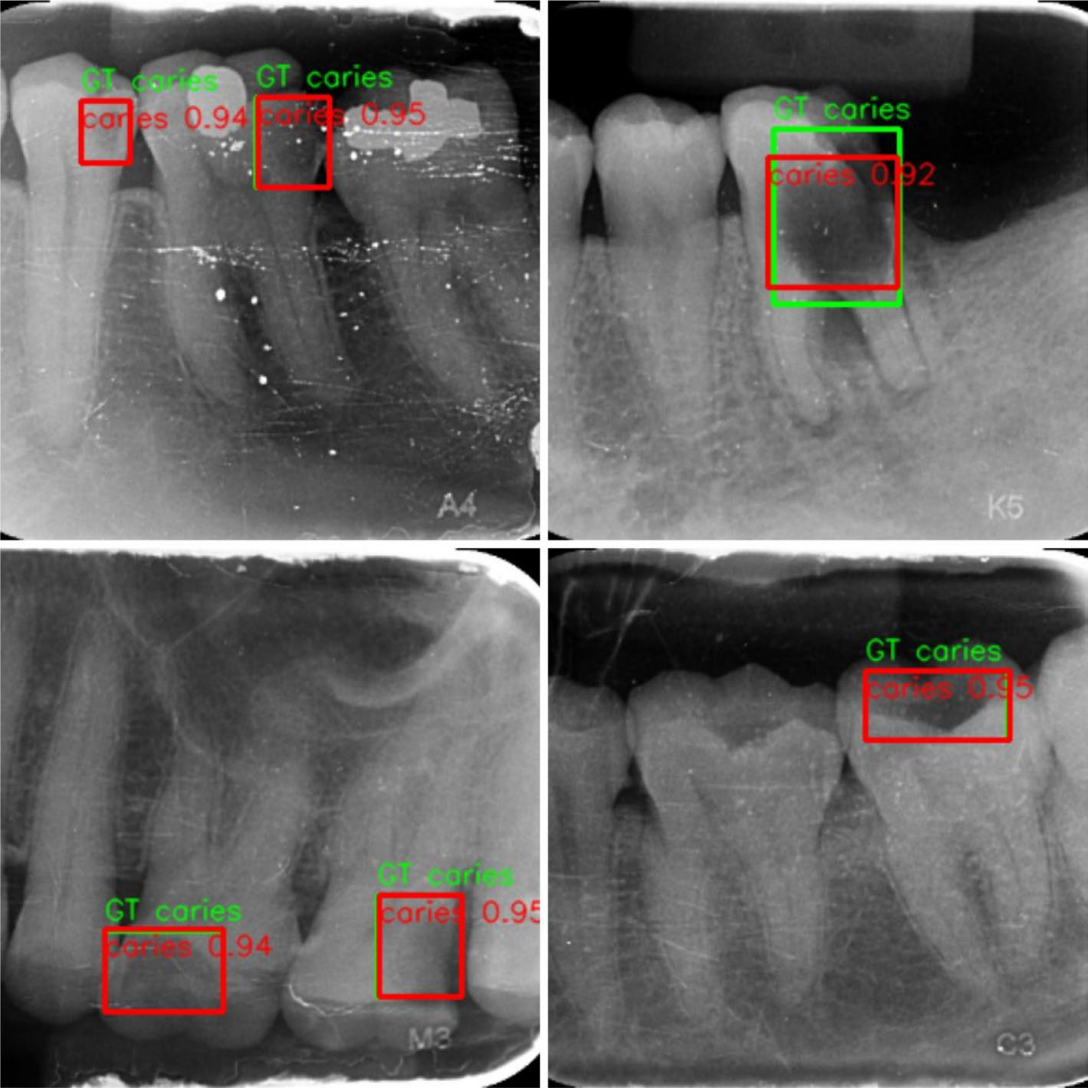
Predicted vs. ground truth caries detection using YOLOv8s+MHSA+GA

Figure 9 shows representative test images with predictions in red and annotations in green. Across varied tooth morphologies and lesion types, the predicted boxes closely overlap the ground truth, indicating precise and well-bounded localization. The consistently high confidence scores and absence of spurious detections on healthy structures visually reinforce the model’s precision and clinical utility.

These qualitative and quantitative results together underscore the model’s reliability and its readiness for real-time clinical use in dental practice, where accuracy and efficiency are both essential. A detailed summary of performance metrics and a head-to-head comparison with baseline and ablated variants are provided in Table 1 In addition, the visual examples demonstrate minimal false positives and tight lesion delineation, supporting precise, confidence-guided decision making at the chairside. Finally, the stable results across varied image appearances and lesion presentations suggest robustness to typical clinical variability, strengthening the case for deployment on routine workflows. For clinicians, this means the system can act as a supportive tool by providing consistent second opinions and highlighting areas that may require closer inspection.

**Table 1:** Comparative performance of the proposed approach and baseline models

Model	Precision	Recall	F1-score	mAP@0.5	mAP@0.5:0.95
YOLOv8s	0.77	0.68	0.72	0.74	0.33
YOLOv8s+MHSA	0.82	0.64	0.72	0.76	0.32
YOLOv8s+GA	0.76	0.64	0.70	0.73	0.30
YOLOv8s+MHSA+GA	0.98	0.94	0.96	0.97	0.94

Precision and Recall were summarized with Wilson 95% Confidence Intervals. The proposed model attains precision 0.98 with a 95% confidence interval from 0.97 to 0.99 and recall 0.94 with a 95% confidence interval from 0.91 to 0.95. Baseline variants are clearly lower: YOLOv8s shows precision 0.77 and recall 0.68, YOLOv8s with GA shows precision 0.76 and recall 0.64, and YOLOv8s with MHSA shows precision 0.82 and recall 0.64. Mean average precision follows the same pattern, with the proposed model reaching 0.97 at IoU 0.5 and 0.94 across 0.5:0.95. These interval estimates in Table 2 demonstrate higher central values and tighter uncertainty for the proposed approach.

**Table 2:** Performance with Wilson 95% Confidence Interval

Model	Precision	$\boldsymbol{P}_{\boldsymbol{L}}$	$\boldsymbol{P}_{\boldsymbol{U}}$	Recall	$\boldsymbol{R}_{\boldsymbol{L}}$	$\boldsymbol{R}_{\boldsymbol{U}}$	mAP@0.5	mAP@0.5:0.95
YOLOv8s	0.77	0.72	0.82	0.68	0.62	0.73	0.74	0.33
YOLOv8s+GA	0.76	0.71	0.81	0.64	0.58	0.70	0.73	0.30
YOLOv8s+MHSA	0.82	0.76	0.86	0.64	0.58	0.70	0.76	0.32
YOLOv8s+MHSA+GA	0.98	0.97	0.99	0.94	0.91	0.95	0.97	0.94

Pairwise comparisons between the proposed detector and each baseline show decisive, consistent gains. Two-proportion tests for precision and recall yield *p ≈ 0.000* in all contrasts, indicating materially higher precision and recall than YOLOv8s, YOLOv8s+GA, and YOLOv8s+MHSA. For precision versus the MHSA variant, $p = 2.22 \times 10^{-16}$. Effect sizes expressed as F1-score improvements: the mean F1-score gain over YOLOv8s is *+0.23* with a *95%* confidence interval from 0.19 to 0.27, over YOLOv8s with GA is *+0.26* with a confidence interval from 0.21 to 0.30, and over YOLOv8s with MHSA is *+0.23* with a confidence interval from 0.19 to 0.28. All intervals lie entirely above zero, confirming statistically meaningful improvements. These outcomes are summarized in Table 3.

**Table 3:** Pairwise significance vs. the proposed model (two-proportion tests)

Baseline Model	Precision *p*-value	Recall *p*-value	$\boldsymbol{\Delta }$F1 (proposed−baseline)	95% CI for $\boldsymbol{\Delta }$F1	Bootstrap *p*-value
YOLOv8s	$0.000\textrm{e}{+00}$	$0.000\textrm{e}{+00}$	*+0.23*	0.19–0.27	$0.000\textrm{e}{+00}$
YOLOv8s+GA	$0.000\textrm{e}{+00}$	$0.000\textrm{e}{+00}$	*+0.26*	0.21–0.30	$0.000\textrm{e}{+00}$
YOLOv8s+MHSA	$2.220\textrm{e}{-16}$	$0.000\textrm{e}{+00}$	*+0.23*	0.19–0.28	$0.000\textrm{e}{+00}$

Over the last training epochs 367–370, both $\textrm{mAP}@0.5$ and $\textrm{mAP}@[0.5{:}0.95]$ showed no significant trend. Spearman’s *ρ = -0.77* with *p = 0.22* for each metric, and the estimated linear slope was approximately $-3.0 \times 10^{-4}$ per epoch with *p = 0.22*. These values indicate a stable performance plateau at convergence, supporting that the reported $\textrm{mAP}$ scores reflect steady generalization rather than a transient fluctuation, as shown in Table 4.

**Table 4:** Training stability of the proposed model

Metric	Spearman’s *ρ *	*p*-value	Linear slope per epoch	*p*-value
mAP@0.5	*-0.77*	0.22	$-3.0 \times 10^{-4}$	0.22
mAP@0.5:0.95	*-0.77*	0.22	$-3.0 \times 10^{-4}$	0.22

### Performance analysis

**Fig. 10:**
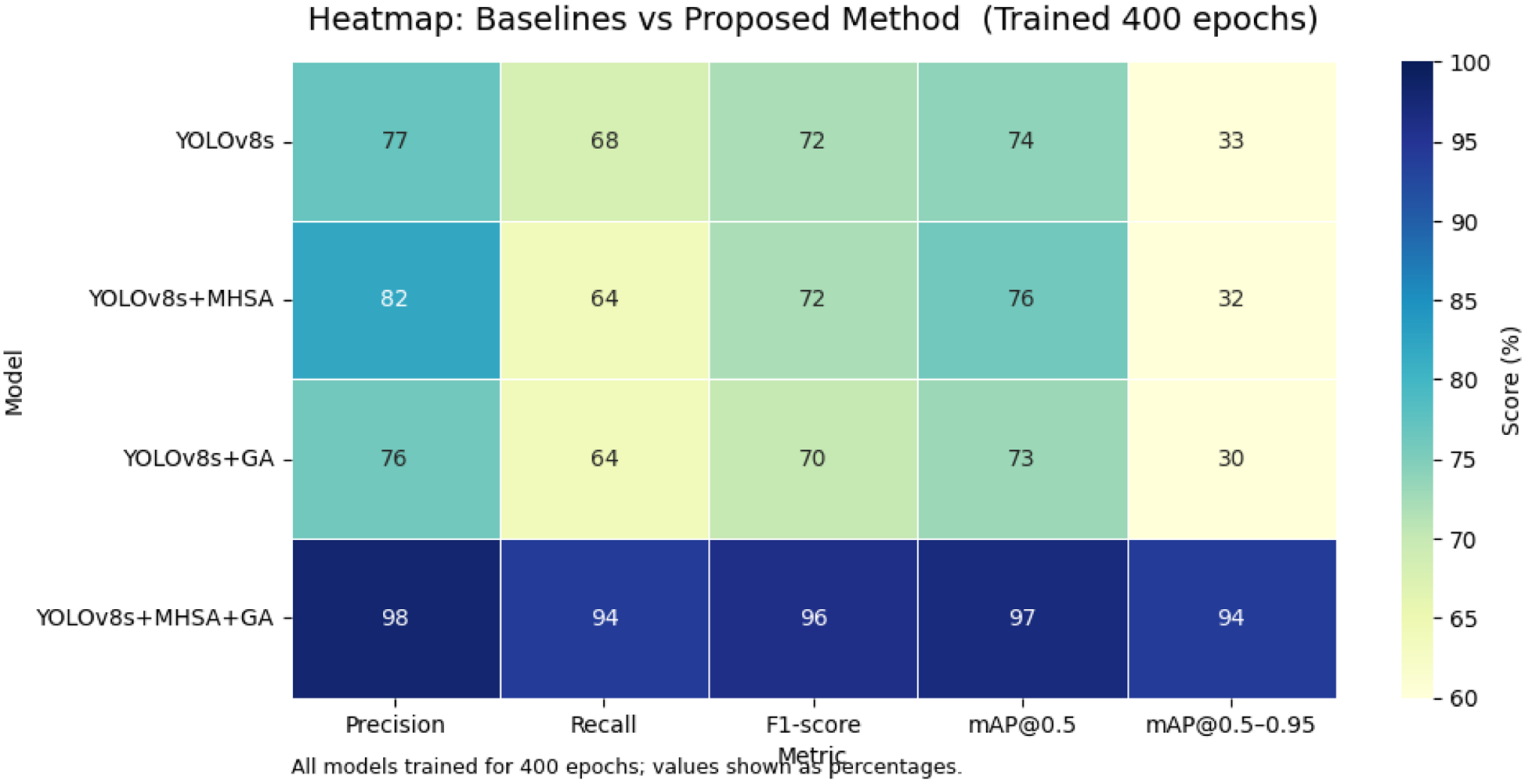
Metric heatmap: proposed vs baselines

Figure 10 visualizes the five-evaluation metrics Precision, Recall, F1 Score, mAP@0.5, and mAP@0.5:0.95—for the YOLOv8 baselines and the proposed attention-augmented, GA-tuned model. Color intensity maps directly to performance, allowing an at-a-glance comparison across rows and columns. The heatmap consistently concentrates the darkest cells in the YOLOv8s+MHSA+GA row, indicating superior results on every metric. This view complements the grouped bars by conveying the same numbers in a compact matrix, making the overall trend unmistakable: adding MHSA and genetic-search optimization yields clear gains in accuracy and reliability, strengthening the case for the proposed configuration in clinical radiograph workflows.

**Fig. 11:**
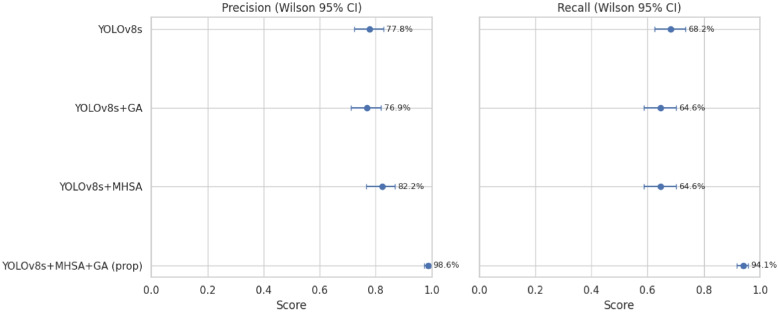
Precision and recall with Wilson 95% CI for all models

**Fig. 12:**
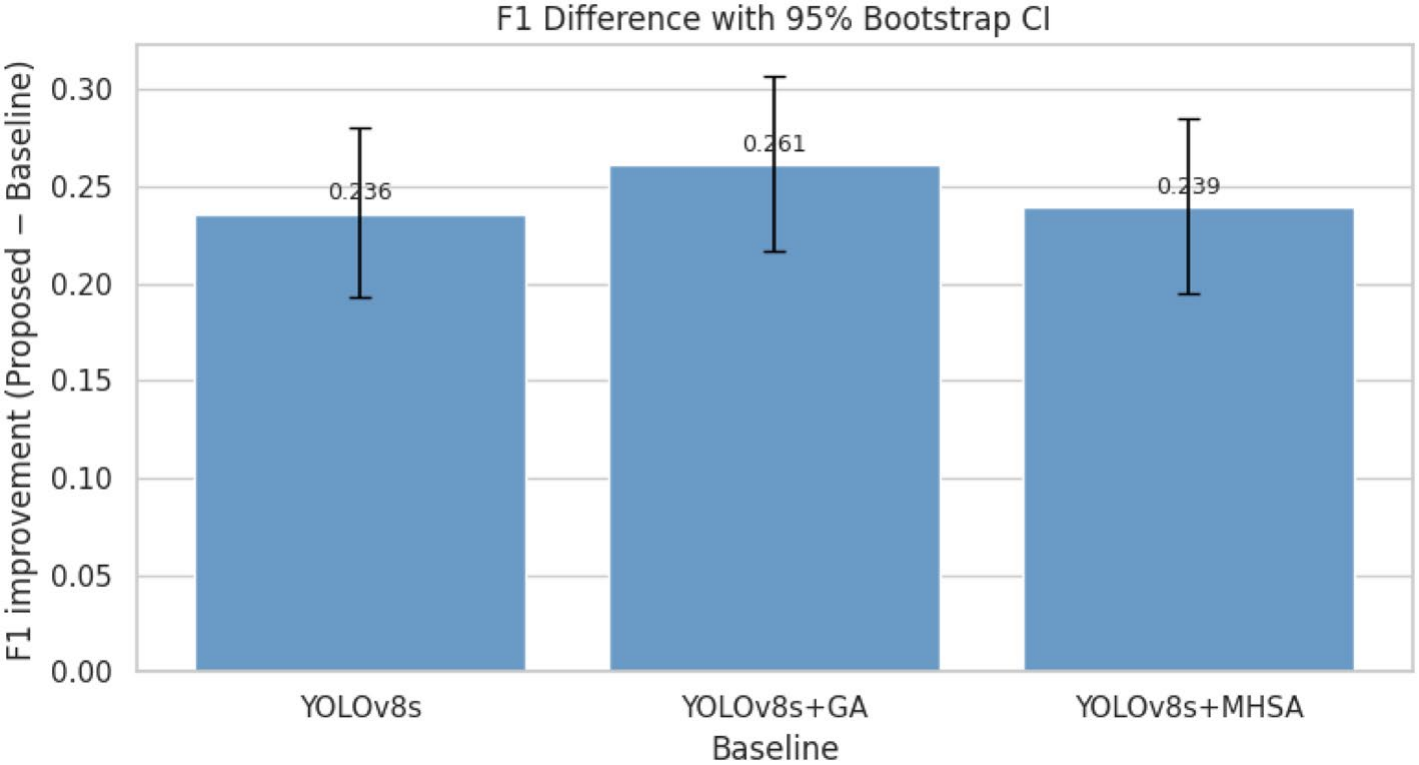
F1 Score improvement of the proposed model over baselines with 95% bootstrap CI

Figure 11 Contrasts Precision and Recall across models using Wilson 95% confidence intervals. The proposed YOLOv8s+MHSA+GA lies far to the right on both axes with tight intervals, indicating very high precision 0.98 and recall 0.94 and low uncertainty. Baselines cluster lower precision and recall with wider intervals. This visualization makes the separation clear while conveying uncertainty for each estimate. Figure 12 Reports effect sizes as the improvement in F1 Score of the proposed model over each baseline, with bootstrap 95% confidence intervals. Gains are substantial and statistically reliable: approximately +0.23 over YOLOv8s, +0.26 over YOLOv8s+GA, and +0.23 over YOLOv8s+MHSA, with all intervals entirely above zero. This Figure provides a direct quantitative summary of the magnitude of improvement and complements the confidence-interval view in Fig. 11.

**Fig. 13:**
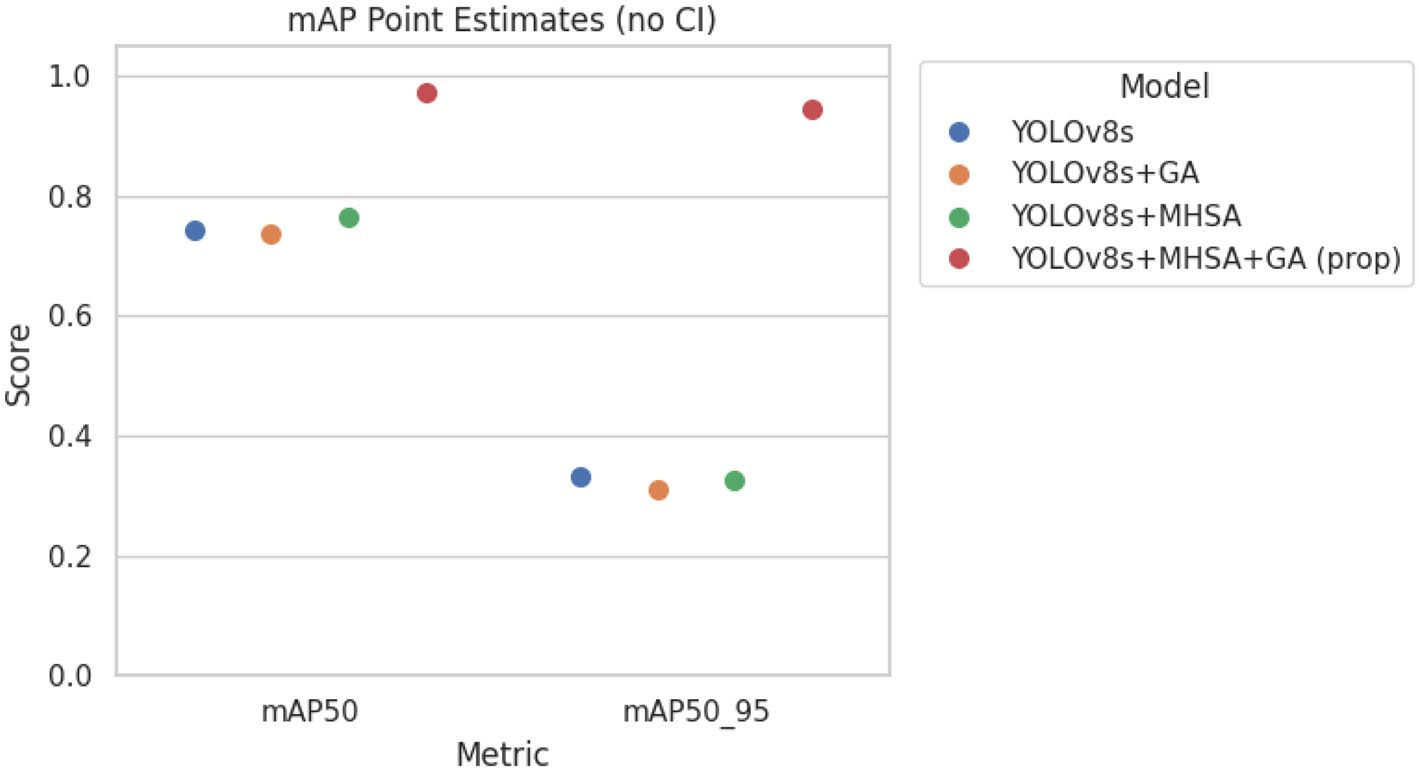
mAP point estimates (IoU 0.5 and 0.5-0.95) for all models

**Fig. 14:**
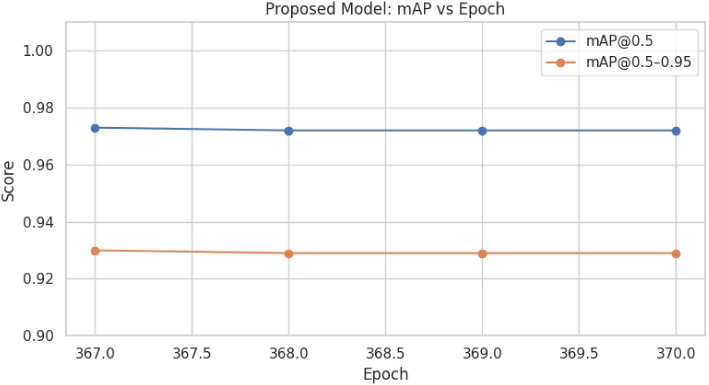
Proposed model mAP vs final epochs showing convergence

Figure 13 shows $\textrm{mAP}$ at IoU *= 0.5* and $\textrm{mAP}@[0.5{:}0.95]$ for each model as point estimates. The proposed detector attains the highest scores on both scales (0.97 and 0.94), while baselines lie around 0.73–0.77 and 0.31–0.33. This plot provides a compact comparison that is consistent with Figs. 11, 12 and Table 2. Figure 14 tracks the proposed model’s $\textrm{mAP}$ over epochs and shows essentially flat curves for both $\textrm{mAP}@0.5$ and $\textrm{mAP}@[0.5{:}0.95]$. Trend statistics confirm no significant change, with Spearman’s *ρ = -0.77* and *p = 0.22*, and a linear slope of approximately $-3.0 \times 10^{-4}$ per epoch with *p = 0.22*. This supports a stable performance plateau at convergence and reduces concerns about epoch-level hand-picking of results.

## Discussion

Accurate localization of small, low-contrast carious lesions on IOPA requires models that balance sensitivity with real-time efficiency. Starting from a convolution-only reference with YOLOv8s, we conducted a set of controlled ablations to isolate the contributions of two factors: first, MHSA inserted into the deeper backbone stages; second, principled schedule selection via a compact GA. Under an identical YOLO-formatted split of 80% training, 10% validation, and 10% testing with a fixed seed of 42 and using gentle radiograph-appropriate augmentations, the reference attained Precision 0.77, Recall 0.68, F1 Score 0.72, mAP at IoU 0.5 of 0.74, and mAP at IoU 0.5:0.95 of 0.33. Adding two lightweight self-attention blocks after the final two C2f stages improved discriminability and ranking, with Precision 0.82, mAP at IoU 0.5 of 0.76, and mAP at IoU of 0.5:0.95 of 0.32 while sensitivity remained similar with Recall 0.64 and F1 Score 0.72. A genetic-algorithm-only control using the baseline backbone and GA-selected hyperparameters produced Precision 0.76, Recall 0.64, F1 Score 0.70, mAP at IoU 0.5 of 0.73, and mAP at IoU of 0.5:0.95 was 0.30 indicating that hyperparameter tuning alone is insufficient when the representational bias remains purely convolutional. In contrast, the combined configuration with YOLOv8s, self-attention, and genetic-algorithm selection achieved Precision 0.98, Recall 0.94, F1 Score 0.96, mAP at IoU 0.5 of 0.97, and mAP at IoU of 0.5:0.95 was 0.94 on the held-out test set. These values represent clinically meaningful gains. The recall increase of 0.26 absolute over the baseline directly targets the most critical failure mode of missed lesions, while the large rise in mAP at IoU 0.5:0.95 from 0.33 to 0.94 demonstrates accurate detections under stricter IoU thresholds and markedly tighter box localization.

Recent radiographic studies report strong lesion-level performance with single-stage YOLO detectors, and attention-augmented backbones have been shown to enhance localization and discrimination of subtle or overlapping structures. Our findings are consistent with these trends. Incorporating deeper-stage attention improved the model’s ability to capture broader contextual information, leading to statistically and clinically meaningful gains in detection and localization performance. These results suggest that both architectural design and training strategy are important considerations for dental radiography tasks.

Self-attention enables the model to integrate global contextual cues that help differentiate carious from non-carious textures in complex radiographic anatomy. While attention alone improved precision and localization quality, the most substantial performance gains were observed when it was combined with schedule optimization through the genetic algorithm. This pattern indicates a complementary effect between contextual feature modeling and stable training dynamics, rather than improvements driven by increased model complexity alone.

At a shared inference setting with a confidence threshold of 0.20 and a NMS IoU 0.60, visual review showed fewer partial boxes and fewer missed proximal lesions with the proposed model, matching the higher Recall and F1 Score. Because the neck and decoupled heads were kept identical across variants, performance gains can be attributed to adding self-attention in the two deepest backbone stages and to schedule/regularization choices discovered by the GA, not to any downstream architectural changes. In our prior work [19] on the same intraoral periapical radiograph dataset, we evaluated a two-stage Faster R-CNN detector for caries detection. Although Faster R-CNN demonstrated reasonable localization capability, it showed lower recall and significantly slower inference compared to the present YOLOv8s framework. These observations motivated the transition to a one-stage detector in the current study. The improved performance and efficiency of YOLOv8s observed here are therefore not based on an isolated architectural choice, but are informed by prior empirical comparison on the same dataset. This supports the suitability of YOLOv8s as a stronger baseline for further architectural enhancements such as MHSA integration and GA-based schedule optimization. When viewed in the context of recent radiographic caries detection studies, the proposed method shows competitive and clinically meaningful performance, particularly in terms of recall, F1-score, and localization quality. Prior studies on bitewing radiographs have reported strong results with YOLO-based, RetinaNet-based, Faster R-CNN, and EfficientDet-based systems, but performance has varied depending on lesion type, image quality, and dataset design. In contrast, the present work focuses on IOPA, which are generally more challenging because of overlapping anatomy and greater projection variability. Therefore, although the proposed YOLOv8s+MHSA+GA framework compares favorably with recent state-of-the-art approaches, direct numerical comparison should be interpreted with caution because of differences in imaging modality, annotation strategy, dataset composition, and evaluation protocols.

Additional evidence from recent literature further supports the importance of robust context modelling for subtle lesion detection. García-Cantu et al. reported clinically useful performance on bitewing radiographs but noted persistent difficulty in identifying very small and early lesions, emphasizing the need for improved localization fidelity and sensitivity [20]. Likewise, Felsch et al. demonstrated that transformer-based architectures can effectively combine localization and classification cues in dental imagery, underscoring the broader value of long-range contextual reasoning in complex diagnostic settings [21].

Earlier dental imaging studies also showed that image variability, artefacts, and preprocessing inconsistencies can substantially affect automated performance. CNN-based tooth detection and classification systems reported encouraging accuracy but remained sensitive to misalignment, noise, and metal artefacts [22–25]. These observations reinforce the importance of designing detectors that are not only accurate under controlled settings but also robust to real-world variability, which was one motivation for combining attention-based context modelling with schedule optimization in the present work.

The dataset is single-center, which may limit generalizability across devices, acquisition protocols, and patient mix. As this study was conducted on a single-center dataset, cross-dataset generalization could not be directly validated. Therefore, the reported performance reflects internal validation under controlled conditions rather than robustness across independent clinical sources. No external validation on public or multi-center datasets was performed in this study due to the limited availability of appropriately annotated IOPA datasets. Labels reflect expert judgment and may embed spectrum bias; multi-reader consensus and per-tooth or per-surface stratification would refine estimates. Evaluation emphasized detection metrics such as mAP at IoU 0.5, mAP at IoU 0.5:0.95, Precision, Recall, and F1 Score; probability calibration, decision-analytic utility, robustness to domain shift and systematic image-quality variation, and deployment considerations such as Picture Archiving and Communication System/Radiology Information System (PACS/RIS) integration, chairside latency were not assessed. Future work will perform external multi-center validation and domain-shift studies across different X-ray machines and acquisition settings, and will investigate efficient adaptation (few-shot fine-tuning/self-training) and continual learning to handle longitudinal drift while avoiding catastrophic forgetting. In addition, lesion-size–stratified analysis was not performed in the present study because the dataset annotations were limited to single-class detection without explicit size-based subgroup labels; future work will incorporate size-aware evaluation to better assess model sensitivity for small and early carious lesions. An additional limitation is that dataset partitioning was performed at the image level rather than the patient level, as consistent patient identifiers were not uniformly available. Consequently, the reported performance should be interpreted with caution, since patient-level partitioning would provide a stricter estimate of generalizability and further reduce potential data leakage. Furthermore, although annotations underwent expert clinical verification, formal inter-observer agreement statistics were not assessed. This should be considered when interpreting the reported performance, and future studies will incorporate multi-expert independent annotation with agreement analysis. Although formal multi-reader annotation and inter-observer agreement were not assessed in this study, the annotations underwent expert clinical verification by a trained clinician with six years of experience in radiographic caries diagnosis. Prior studies have reported that AI systems for caries detection generally achieve performance comparable to intermediate-to-senior clinicians rather than replacing expert judgment. Accordingly, the proposed system is intended to function as a decision-support tool to assist practitioners in radiographic interpretation, rather than as a substitute for clinical expertise. Future studies will incorporate multi-reader independent annotation and reader–AI performance comparison to further establish clinical reliability.

## Conclusion and future work

In this study, we proposed an enhanced YOLOv8s-based framework incorporating deep-stage MHSA and GA–based schedule optimization for automated caries detection on IOPA. The proposed method demonstrated improved performance in terms of precision, recall, F1 Score, and localization accuracy compared to baseline configurations under a consistent evaluation protocol. These results suggest that integrating global contextual modelling with optimized training strategies can enhance detection performance for subtle and low-contrast lesions. While the findings are promising, further validation on diverse datasets and clinical settings is necessary to fully establish generalizability and real-world applicability. Such systems have the potential to assist clinicians in improving diagnostic consistency and early detection, particularly in settings with limited specialist availability.

Future work will focus on validating the proposed model on external public datasets and multi-center radiographic cohorts to further assess its generalizability. We will also perform lesion-size–stratified evaluation to assess the model’s sensitivity for detecting small, medium, and large carious lesions, with particular emphasis on early-stage lesions. To ensure continued relevance with newer YOLO generations, we will port and re-evaluate the same two components—deep-stage MHSA insertion and GA-based schedule selection—on more recent detector families (e.g., Ultralytics YOLO11/YOLO12 and recent YOLOv13-style architectures) and report whether the same gains persist under identical data splits and operating points. A multi-reader annotation study with consensus will quantify inter-observer variability and mitigate spectrum bias. Beyond detection metrics, we will assess probability calibration (ECE, NLL) and decision-analytic utility. Robustness will be tested under domain shift and controlled degradations (noise, blur, compression), with per-tooth/per-surface analyses and error taxonomies to reveal failure modes. For deployment, we will profile end-to-end inference latency, explore model compression (quantization, pruning, distillation), and prototype PACS/RIS integration in our future work. To further handle domain shift across X-ray devices and acquisition settings, we will investigate domain adaptation and test-time adaptation approaches, and evaluate few-shot/meta-learning style updates that can personalize the detector using a small number of labeled images from a new clinic. We will also explore continual learning with drift monitoring to keep the model up to date under changing protocols while avoiding catastrophic forgetting.

## Data Availability

The datasets generated and/or analysed during the current study are not publicly available due to institutional and patient privacy restrictions under the terms of the ethical approval granted by the Sibar Institute of Dental Sciences (SIDS), but are available from the corresponding author on reasonable request and with permission of the institution.

## Abbreviations

AI: Artificial Intelligence
DL: Deep Learning
CNN: Convolutional Neural Network
MHSA: Multi-Head Self-Attention
GA: Genetic Algorithm
IOPA: Intraoral Periapical Radiograph
FPN: Feature Pyramid Network
PAN: Path Aggregation Network
NMS: Non-Maximum Suppression
mAP: Mean Average Precision
IoU: Intersection over Union
ECE: Expected Calibration Error
NLL: Negative Log-Likelihood
PACS: Picture Archiving and Communication System
RIS: Radiology Information System
